# Ultrafast, Broadband Photodetector Based on MoSe_2_/Silicon Heterojunction with Vertically Standing Layered Structure Using Graphene as Transparent Electrode

**DOI:** 10.1002/advs.201600018

**Published:** 2016-07-05

**Authors:** Jie Mao, Yongqiang Yu, Liu Wang, Xiujuan Zhang, Yuming Wang, Zhibin Shao, Jiansheng Jie

**Affiliations:** ^1^ Institute of Functional Nano and Soft Materials (FUNSOM)Collaborative Innovation Center of Suzhou Nano Science and Technology (Nano‐CIC)Jiangsu Key Laboratory for Carbon‐Based Functional Materials and DevicesSoochow UniversitySuzhouJiangsu215123P. R. China; ^2^ School of Electronic Science and Applied PhysicsHefei University of TechnologyHefeiAnhui230009P. R. China

**Keywords:** broadband photodetector, graphene electrode, heterojunction, MoSe_2_, silicon

## Abstract

**A MoSe_2_/Si heterojunction photodetector** is constructed by depositing MoSe_2_ film with vertically standing layered structure on Si substrate. Graphene transparent electrode is utilized to further enhance the separation and transport of photogenerated carriers. The device shows excellent performance in terms of wide response spectrum of UV–visible–NIR, high detectivity of 7.13 × 10^10^ Jones, and ultrafast response speed of ≈270 ns, unveiling the great potential for the heterojunction for high‐performance optoelectronic devices.

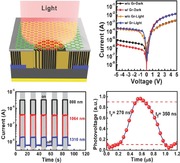

2D materials such as graphene, hexagonal boron nitride (h‐BN), VS_2_, Bi_2_S_3_, and GaSe have been widely studied, because of their great potential in the field of catalysis, microelectronics, ion storage, and optoelectronics.^1^, ^2^, ^3^, ^4^, ^5^, ^6^, ^7^, ^8^, ^9^, ^10^ As one of the most significant members of 2D materials family, transition metal dichalcogenides (TMDs), such as MoS_2_, MoSe_2_, WS_2_, and WSe_2_, have attracted tremendous attention currently due to their outstanding electronic, optical, and mechanical properties.^11^, ^12^, ^13^, ^14^, ^15^, ^16^, ^17^, ^18^, ^19^ Monolayer MoSe_2_ is a sandwich structure consisting of one Mo atom and two Se atoms, and the different layers are interacted by van der Waals force.^20^, ^21^ Like MoS_2_, the properties of MoSe_2_ are also related with layer numbers; monolayer MoSe_2_ exhibits a direct bandgap of 1.6 eV, whereas it changes into indirect bandgap of 1.1 eV for bulk or multilayer MoSe_2_.^22^, ^23^ N‐type channel behavior with an average mobility of ≈50 cm^2^ V^−1^ s^−1^ has been investigated for field‐effect transistors (FETs) based on monolayer MoSe_2_,^24^ while for bulk MoSe_2_ the carrier mobility is ≈100 cm^2^ V^−1^ s^−1^.^25^ In comparison with MoS_2_, MoSe_2_ shows a stronger light absorption in the solar spectrum range.^26^ It has been proved that MoSe_2_ can absorb nearly 5%–10% of incident sunlight in a thickness less than 1 nm.^26^ Moreover, bandgap engineering on MoSe_2_ could be accomplished by forming ternary alloy of MoS_2(1–*x*)_Se_2*x*_.^17^, ^27^, ^28^ Owing to these appealing properties, many efforts have been devoted to exploit the applications of MoSe_2_ in diverse fields, including ion batteries, FETs, and photovoltaics, etc.^29^, ^30^, ^31^, ^32^, ^33^

Photodetectors based on 2D materials have been intensively investigated in recent years.^34^, ^35^, ^36^, ^37^ They show great potential for broadband, high‐sensitivity, and flexible photodetection due to the large light absorption and ultrathin thickness.^34^, ^38^ Although the gapless band structure of graphene offers the capability of ultrawide band detection, the short lifetimes of the photogenerated carriers in graphene (in the ps range) hinder the improvement of photocurrent.^36^, ^39^, ^40^ In comparison to graphene, TMDs such as MoS_2_ and MoSe_2_ possess large bandgap and thus higher carrier lifetimes, making them as promising candidates for high‐sensitivity photodetectors.^34^, ^41^, ^42^ For a practical photodetector, fast response speed is particularly important for the applications such as imaging and optical communication. However, due to the difficulty in controllable doping, most of the TMDs‐based photodetectors have a lateral device structure of metal‐semiconductor‐metal (MSM) or phototransistor. The transit time of carriers between two contact electrodes limits the device response speed.^34^ Moreover, the mono‐/multilayer structure of the nanosheets makes them very susceptible to the surface contamination or molecule adsorption, which also degrades the respond speed due to the carrier trapping/detrapping process.

To date, a few works have been reported for the photodetectors based on mono‐/multilayer MoSe_2_ nanosheets.^43^, ^44^, ^45^ Although they show outstanding device characteristics such as remarkable light response, their response speed (15 ms–8 s) remains too low to meet the requirements for practical applications, and broadband light detection is yet to be demonstrated.^43^, ^44^, ^45^ In conventional photodetectors, diode structure with vertical p–n junctions are normally adopted. Therefore response speed could be greatly enhanced due to the presence of strong built‐in electric field at junction interface as well as the shorter carrier transit time.^46^, ^47^, ^48^ In light of this, fabrication of MoSe_2_ homo‐/heterojunction‐based photodetectors is much desirable to further boost their performance. For instance, Duan et al. demonstrated the lateral epitaxial growth of MoSe_2_/MoS_2_ heterojunctions, which showed pronounced photoresponse characteristics.^49^ Choi et al. also reported the construction of MoSe_2_/graphene van der Waals heterostructures, and investigated the rapid transfer of photogenerated charge carriers between MoSe_2_ and graphene.^50^

Herein, we demonstrated the fabrication of MoSe_2_/Si heterojunctions for high‐speed, broadband response photodetectors. High‐quality p‐n heterojunctions were formed by combing the p‐type Si substrate with n‐type MoSe_2_ film, thus bypassing the difficulty in controllable doping of MoSe_2_. More importantly, the MoSe_2_ film possessed a unique vertically standing layered structure, enabling the fast separation and transport of photogenerated carriers. Graphene (Gr) transparent electrode was adopted to further enhance the carrier collection and consequently reduce the recombination at junction interface. Significantly, the Gr/MoSe_2_/Si heterojunction photodetector exhibited a wide photoresponse range of 350–1310 nm and an extremely fast response speed of ≈270 ns, which represent the best values reported thus far for MoSe_2_ or MoS_2_ based photodetectors (**Table** **1**).^43^, ^44^, ^45^, ^51^, ^52^, ^53^, ^54^ It is expected that the MoSe_2_/Si heterojunctions will have important applications for high‐performance optoelectronic devices.

**Table 1:** Performance comparison of our Gr/MoSe_2_/Si heterojunction‐based photodetector with other photodetectors in literatures

Devices	Wavelength [nm]	Detectivity [Jones]	Pulsed light frequency [Hz]	Rise time [μs]	Fall time [μs]	References
Gr/MoSe_2_/Si heterojunction	365–1310	7.13 × 10^10^	10^6^	0.27	0.35	This work
Monolayer MoSe_2_ phototransistor	532, 650	_	_	2.5 × 10^4^	2.5 × 10^4^	43
MoSe_2_ nanosheets photoconductor	650	_	_	2.9 × 10^6^	4.6 × 10^6^	44
Monolayer MoSe_2_ photoconductor	532	_	_	6 × 10^4^	6 × 10^4^	45
MoSe_2_ nanostructure photoconductor	650	_	_	7.9 × 10^6^	9.8 × 10^6^	51
Multilayer MoSe_2_ phototransistor	532	_	_	1.5 × 10^4^	3 × 10^4^	52
Multilayer MoS_2_ phototransistor	532	≈10^10^	_	70	110	53
Gr/Si heterojunction	400‐900	2.1 × 10^8^	20	1.2 × 10^3^	3 × 10^3^	54

In this study, MoSe_2_ films with vertically standing layered structure were prepared by magnetron sputtering method with a thickness of ≈200 nm (see Figure S1 in the Supporting Information). The scalable sputtering method ensures the large‐area fabrication of high‐quality MoSe_2_ films with high uniformity, in contrast to the small size MoSe_2_ nanosheets obtained by exfoliation or thermal evaporation methods.^55^, ^56^ The as‐prepared MoSe_2_ film has trigonal phase (see Figure S2 in the Supporting Information). After deposition, the film was annealed in a rapid thermal processing systerm (RTP) at 800 °C in Ar for 20 min to further improve its crystal quality. **Figure** **1**a depicts the Raman spectra of the MoSe_2_ films of both as‐prepared and annealed MoSe_2_ films. Two typical Raman active modes, A_1g_ and E_2g_, for MoSe_2_ are observed. The prominent A_1g_ mode relates to the out‐of‐plane vibration of Se atoms, while the E_2g_ mode is associated with the in‐plane vibration of Mo and Se atoms (see the inset in Figure 1a). Prior research results indicate that the change in layer number of MoSe_2_ nanosheets will cause a significant difference in the locations of scattering modes in Raman spectra; the E_2g_ vibration will redshift, whereas the A_1g_ vibration will blueshift, with increasing MoSe_2_ layer number.^20^, ^57^ Considering the large thickness of the MoSe_2_ film in this work, its Raman spectra are more likely to be identical to that of the MoSe_2_ bulk material or multilayer nanosheets. From Figure 1a, A_1g_ and E_2g_ scattering modes located at wave numbers of 240.3 and 288.1 cm^−1^, respectively, can be identified for the annealed film, which is consistent with previous reports for MoSe_2_ film.^58^, ^59^ It is reported that the obvious E_2g_ peak usually occurs for monolayer MoSe_2_ nanosheet, whereas a thick MoSe_2_ film (≥2 layers) often exhibits a weak or invisible E_2g_ peak.^59^, ^60^ Presumably, the unusually high E_2g_ peak for the sputtering fabricated MoSe_2_ film may be attributed to the unique vertically standing layered structure.^61^ Figure 1b reveals an obvious movement of A_1g_ peak from 235.2 cm^−1^ before annealing to 240.3 cm^−1^ after annealing, along with the decrease of peak width upon annealing. It is known that the A_1g_ peak location of 240.3 cm^−1^ is more close to the standard value for high‐quality MoSe_2_ (240.6 cm^−1^).^58^, ^59^ Therefore, the Raman spectra clearly show that the film quality is significantly improved after annealing treatment. The components of the annealed MoSe_2_ film were further studied by X‐ray photoemission spectroscopy (XPS), as shown in Figure 1c,d. The Mo 3d shows two peaks at 229.2 and 232.3 eV, which can be attributed to the Mo 3d_5/2_ and Mo 3d_3/2_, respectively, confirming the existence of Mo^4+^.^58^, ^62^, ^63^ The peaks at 54.8 and 55.6 eV are attributed to the doublet Se 3d_5/2_ and Se 3d_3/2_, respectively, corresponding to the divalent selenide ions (Se^2−^).^58^, ^62^, ^63^

**Figure 1:**
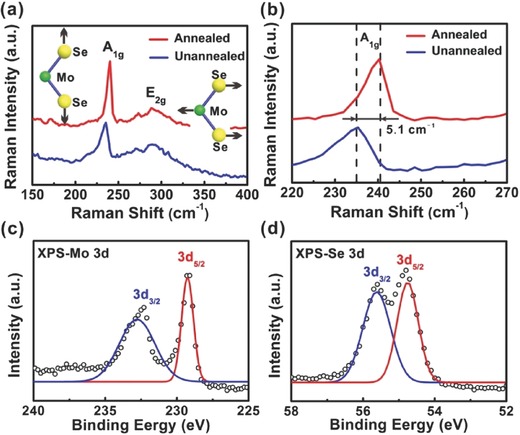
a) Raman spectra of both as‐prepared and annealed MoSe_2_ films. Schematic illustrations in (a) depict the atomic vibration direction of A_1g_ (left) and E_2g_ (right) Raman modes of MoSe_2_ film. b) Comparison of the A_1g_ peaks in Raman spectra for the as‐prepared and annealed MoSe_2_ films. XPS spectra show the binding energies of c) Mo and d) Se for the annealed MoSe_2_ film.

**Figure** **2**a illustrates the device structure of MoSe_2_/Si heterojunction photodetector. MoSe_2_ film was first deposited on p‐type Si substrate with a predefined SiO_2_ circular window (*d* = 3 mm) by magnetron sputtering, which determines the effective device area. Ag electrode (50 nm) was deposited around the window as the top ohmic contact to MoSe_2_ film (see Figure S3 in the Supporting Information). Then three‐layer graphene film with sheet resistance of ≈380 Ω sq^–1^ (conductivity ≈7740 S/cm) was transferred to the top of MoSe_2_ film as transparent electrode. The use of graphene electrode ensures the efficient light absorption of MoSe_2_/Si heterojunction, meanwhile facilitates the transport of photogenerated carriers from MoSe_2_ film to the Ag top electrode. Afterward, Au electrode (50 nm) was deposited at the rear side of p‐type Si as back ohmic contact. To gain more insight into the structure of the MoSe_2_/Si heterojunction, cross‐sectional transmission electron microscopy (TEM) investigation was performed, as shown in Figure 2b‐e. The MoSe_2_ film deposited by sputtering has a uniform thickness of ≈200 nm (Figure 2b). EDS elemental mappings on Si, Mo, and Se at the red square area in Figure 2b prove the formation of MoSe_2_/Si heterojunction. The uniform colour contrast for Mo and Se elements is an evidence of the high uniformity of the MoSe_2_ film. The energy dispersive X‐ray spectroscopy (EDS) line‐scanning analysis, Figure 2c, further confirms the compositions of MoSe_2_/Si heterojunction. From the high‐resolution TEM (HRTEM) image at the junction interface (Figure 2d), we can see that there is a thin interfacial oxide layer (≈5 nm) formed between the Si substrate and the MoSe_2_ film. Close investigation on the MoSe_2_ film, Figure 2e, discloses the distinct vertically standing layered structure of the film, i.e., the growth direction of the (001) molecule planes of MoSe_2_ are perpendicular to the Si substrate, in contrast the parallel growth of the MoSe_2_ nanosheets fabricated by conventional chemical vapour deposition (CVD) method.^57^, ^64^ The dimension of individual crystal grains in the MoSe_2_ film is 4–6 nm wide, corresponding to about 6–9 MoSe_2_ monolayers (Figure 2e). As one of the remarkable characteristics of 2D materials, they show pronounced anisotropic conduction in the in‐plane direction and out‐of‐plane direction. The weak van der Waals force and the large layer distance between adjacent layers lead to much lower out‐of‐plane electrical and thermal conductivities compared to those of their in‐plane analogs.^65^, ^66^ In this regards, the vertically standing layered structure endows the MoSe_2_ film distinct electrical properties with much efficient carrier transport from the junction interface to the top electrode.

**Figure 2:**
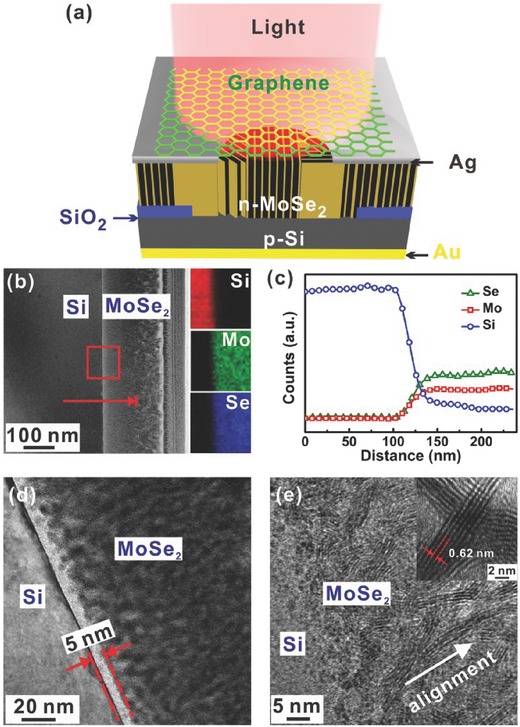
a) Schematic illustration of the Gr/MoSe_2_/Si heterojunction photodetector with graphene transparent electrode. b) Cross‐sectional TEM image of the interface of MoSe_2_ and Si. Insets show the EDS element mappings of Si, Mo, and Se at the red square area. c) The line‐scan EDS analysis along the red line from Si to MoSe_2_ film in (b). d) HRTEM image of the heterojunction, indicating the existence of an interfacial oxide layer (≈5 nm). e) HRTEM image of the MoSe_2_ film, verifying the vertically standing layered structure of the film. Inset shows the enlarged HRTEM image. The distance between two layers is ≈0.62 nm, corresponding to the (001) face of MoSe_2_.

**Figure** **3**a,b depicts the current versus voltage (*I*–*V*) characteristics of the MoSe_2_/Si heterojunctions in the dark and under light illumination, respectively. Both of the devices with and without graphene transparent electrode were measured to ascertain the role of graphene in determining the device performance. From the dark *I*–*V* curves, we note that the dark current at reverse bias direction (−5 V) decreases from 9.35 μA to 2.82 μA, whereas the dark current at forward bias direction (+5 V) increases from 2.6 mA to14.9 mA, after the use of three‐layer graphene transparent electrode. Correspondingly, the rectification ratio improves significantly from 278 for the device without graphene to 5284 for the device with graphene within ±5 V. As a result of the improved diode characteristic, under white light illumination (23 mW cm^−2^), the photocurrent at −5 V increases remarkably from 202 μA to 263 μA after the use of graphene transparent electrode. Figure 3b depicts the magnified *I*–*V* curves around the zero point under light illumination, revealing an open circuit voltage (*V*_oc_) of 100 mV for the device with graphene electrode, in contrast to the much lower *V*_oc_ of 40 mV for the device counterpart without graphene electrode. Ideality factor (*n*) of the MoSe_2_/Si heterojunctions were deduced from the slops of the semilog *I*–*V* curves at the forward bias direction (Figure 3c), according to the following equation^67^

(1) $$n\;=\frac{q}{k_{B}T}\;\frac{dV}{d\ln\;I}$$

where *q* is the unit charge, *k*_B_ is the Boltzmann's constant, and *T* is the absolute temperature. The ideality factor of the MoSe_2_/Si heterojunction without graphene electrode exhibits a larger value of 2.43, compared to that of 1.79 for the device with graphene electrode. The above results collectively demonstrate that the graphene electrode plays a crucial role in enhancing the device performance of MoSe_2_/Si heterojunction. To understand the superior performance of MoSe_2_/Si heterojunction with graphene transparent electrode, Figure 3d,e illustrates the energy band alignments of the MoSe_2_/Si heterojunctions without and with graphene electrode, respectively. The n‐type MoSe_2_ film will form type II heterojunction with p‐type Si substrate, allowing the efficient separation of photogenerated electron‐hole pairs at junction interface. Under light illumination, electrons inject into the n‐type MoSe_2_ film, while holes inject into p‐type Si, forming the photocurrent. Notably, the introduction of graphene at MoSe_2_ side can greatly enhance the separation of photogenerated carriers. Moreover, the high conductivity of graphene can ensure the fast transport of electrons to the outside electrical circuit. As a result, the accumulation of electrons in MoSe_2_ film is avoided. This further reduces the electron–hole recombination and contributes to the improved photocurrent of the MoSe_2_/Si device. The zero bias barrier of the heterojunction, *Φ*_b_, was calculated based on following equation^68^

(2) $$I\;=I_{S}\;\left[\exp\left(\frac{eV}{nk_{B}T}\right)-1\right]$$

**Figure 3:**
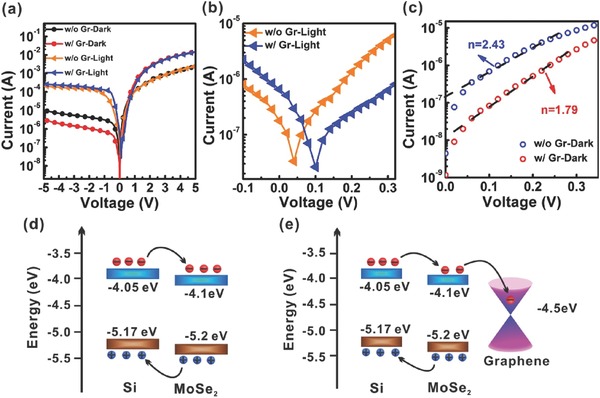
a) Typical *I*–*V* curves of the MoSe_2_/Si heterojunction with and without graphene transparent electrode measured in dark and under white light illumination (23 mW cm^−2^), respectively. b) Magnified *I*–*V* curves around zero point under light illumination. c) Semilogarithmic *I*–*V* curve at forward bias of 0–0.4 V in the dark. The semilogarithmic curves could be fitted by straight lines. Energy band alignments of the MoSe_2_/Si heterojunctions d) without graphene and e) with graphene transparent electrode. The conduction band minimum (CBM) and the valence band maximum (VBM) of Si and MoSe_2_ are represented in blue colour and brown colour, respectively. The work function of graphene (4.5 eV) is also shown in black line. The energy scale is referenced to the vacuum level.

The saturation current of *I*_S_ is defined by

(3) $$\;I_{S}=\;AA^{*}T^{2}\exp\left(-\frac{q\Phi_{b}}{k_{B}T}\right)$$

where *A* is the area of the device (7.06 mm^2^), A* is the effective Richadson constant, and it is 32 A cm^−2^ K^−2^ for p‐type Si.^69^ Based on above equations, *Φ*_b_ is deduced to be 793 mV for the heterojunction device with graphene top electrode. The large bulit‐in electrical field is responsble for the effective spearation of photogenerated carriers.

To evaluate the performance of the Gr/MoSe_2_/Si heterojunction for broadband detection, UV light (365 nm), green light (500 nm), and red light (650 nm) were chosen as exciting light sources. From *I*–*V* curves of the device measured at different light wavelengths (**Figure** **4**a), it is observed that the device shows a remarkable photocurrent upon incident light illumination at reverse bias. The high sensitivity at reverse bias is due to the fact that the photogenerated electron‐hole pairs significantly change the concentration of minority carriers, which dominates the photocurrent under a reverse bias.^70^ From the time‐dependent photocurrent excited by pulsed UV light (365 nm, 15 mW cm^−2^) at different reverse bias voltages, Figure 4b, we note that the device exhibits a high sensitivity to the UV light with a large *I*_on_/*I*_off_ ratio of 140 and 130 at −1 V and −2 V, respectively. The fast response and recovery speed can be deduced by the steep rise and fall edges of the response curves, indicating the effective generation and separation of electron–hole pairs in the Gr/MoSe_2_/Si heterojunction. In addition, excellent stability and reproducibility can also be verified by the unchanged response current of the device illuminated with a pulsed light with about 16 s per cycle. To quantify the performance of the Gr/MoSe_2_/Si photodetector, two key figure‐of‐merit parameters, i.e., responsivity (*R*) and detectivity (D*) that indicate the efficiency of a detector responding to optical signals and the ability of a detector to detect weak optical signals, respectively, are calculated by following equations^71^

(4) $$R\;=\frac{I_{ph}}{P_{in}}$$

(5) $$\;D^{*}=\frac{A^{1/2}R}{{\left(2qI_{d}\right)}^{1/2}}\;=\frac{R}{{\left(2qJ_{d}\right)}^{1/2}}$$

where *I*_ph_, *P*_in_, *I*_d_, and *J*_d_ represent the photocurrent, incident light power, dark current, and dark current density, respectively. Figure 4c plots both the responsivity and detectivity at different light wavelengths as a function of applied reverse bias voltage. It is seen that the responsivity at different wavelengths increases, whereas the detectivity first increases and then decreases, with increasing reverse bias voltage. The higher reverse bias can result in a larger photocurrent under the same incident light intensity since more carriers can pass through the junction, thereby contributing to an increased responsivity. However, the dark current will increase at the meantime, ultimately resulting in the decrease of detectivity at high reverse bias. Based on Equations (4) and (5), the Gr/MoSe_2_/Si photodetector shows optimal *R* and D* values of 270 mA W^−1^ and 7.13 × 10^10^ Jones (Jones = cm Hz^1/2^ W^−1^) at 650 nm, 93 mA W^−1^ and 2.55 × 10^10^ Jones at 500 nm, and 182 mA W^−1^ and 6.22 × 10^10^ Jones at 365 nm, respectively. Figure 4d depicts the absorption spectrum of the MoSe_2_/Si heterojunction, with the spectra of both of MoSe_2_ film on quartz substrate and bare Si substrate for comparison. The MoSe_2_ film possesses stronger light absorption at both short wavelength direction (<800 nm) and long wavelength direction (>1060 nm) as compared to the bare Si substrate. Therefore, the combination of MoSe_2_ film with Si substrate makes the system capable of absorption of the broadband light ranging from UV, visible, to NIR light. In addition, it is noted that there is an absorption valley around 500 nm. This may be responsible for the relatively lower responsivity and detectivity at that wavelength.

**Figure 4:**
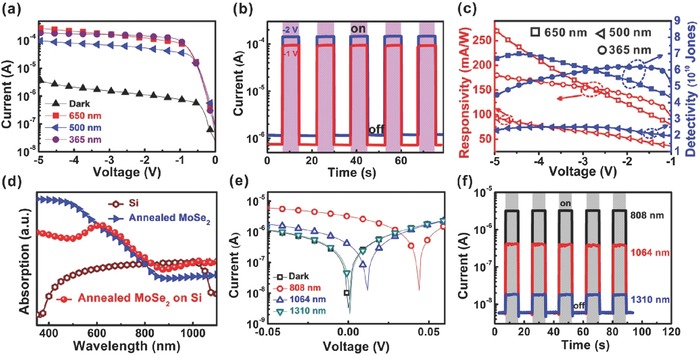
a) *I*–*V* characteristics of the Gr/MoSe_2_/Si photodetector measured in the dark and under light illumination with varied wavelengths of 365, 500, and 650 nm, respectively. Three‐layer graphene was used as the transparent electrode. The light intensities of the light sources were fixed at 15 mW cm^−2^. b) Time‐dependent photocurrent excited by pulsed light at 365 nm (15 mW cm^−2^). Different bias voltages of −1 V and −2 V were applied. c) Plots of responsivity and detectivity of the device at varied light wavelengths as a function of applied reverse bias. d) Absorption spectrum of the MoSe_2_ film on Si substrate. The absorption spectra of MoSe_2_ film grown on quartz substrate under the same conditions and bare Si substrate were also plotted for comparison. e) *I*–*V* characteristics of the device measured in the dark and under laser illumination with different wavelengths of 808, 1064, and 1310 nm, respectively. The light intensity was maintained at 15 mW cm^−2^. f) Photoswitching curves of the device in response to pulsed light illumination with various wavelengths at a bias voltage of 0 V.

The NIR photoresponse of the Gr/MoSe_2_/Si device was further assessed by using the 808, 1064, and 1310 nm lasers as light sources. In Figure 4e, the device exhibits remarkable photoresponse to 808 and 1064 nm lasers. However, photoresponse becomes weaker for the 1310 nm laser since its energy (0.95 eV) is already smaller than the bandgap of MoSe_2_ film (1.1 eV). Significantly, owing to the pronounced photovoltaic behavior, the device can operate at zero bias voltage with excellent reproducibility and stability (Figure 4f). The step rise and fall edges to pulsed light also reveal that the photocurrent and photo‐induced voltage are originated from photovoltaic effect, instead of bolometric effect.

Fast response of a photodetector is requisite for many advanced applications such as optical communication, biological sensing, missile tracking, and so on. In this work, we further investigated response speed of the Gr/MoSe_2_/Si heterojunction photodetector by using an oscilloscope to monitor variation of photovoltage under pulsed red light illumination (**Figure** **5**a). The pulsed red light (650 nm) was produced from a laser diode (LD) supplied by a tuneable square‐wave signal generator at 14 V. Figure 5b,c and Figure S4 in the Supporting Information show the photoresponse of the Gr/MoSe_2_/Si photodetector to pulsed light with frequency ranging from 1 kHz to 1 MHz, manifesting that the device can work well even for high frequency (MHz) optical signals. In addition, from the enlarged photoresponse curve at 1 MHz, Figure 5d, rise time (*t*_r_) and fall time (*t*_f_) are estimated to be 270 and 350 ns, respectively. The response time of the LD light source is ≈6 ns (Figure 5d), ensuring the accurate evaluation of the device response time. It is noteworthy that this response speed is much quicker than the current reports on MoSe_2_‐based photodetectors, and even faster than other MoS_2_‐ or graphene‐based photodetectors (Table 1). This ultrafast response speed can be attributed to the unique device structure of the Gr/MoSe_2_/Si photodetector: (i) Unlike the conventional photoconductors or phototransistors with MSM structures, whose response speed is usually limited by the transmit time of the carriers between two contacts and the defects/traps in the conduction channels, strong built‐in electric field in the Gr/MoSe_2_/Si heterojunction photodetector can greatly facilitate the separation and transport of photogenerated carriers. (ii) The distinct vertically standing layered structure of MoSe_2_ film ensures the fast transport of photogenerated carriers along the vertical direction due to the high in‐plane mobility. The transmit time should be less than 1 ns from the junction interface to the top electrode by assuming a mobility of 100 cm^2^ V^−1^ s^−1^ and a layer thickness of 200 nm for the MoSe_2_ layer. (iii) The use graphene transparent electrode can further enhance the carrier collection and thus reduce the carrier recombination. This is also evidenced by Figure 5e, in which the temporal responses of both the devices with and without graphene electrodes were measured. The former exhibits much faster light response than the latter. Furthermore, we studied the relative balance (V_max_–V_min_)/V_max_ as a function of pulsed light frequency (Figure 5f). It is noteworthy that relative balance of the Gr/MoSe_2_/Si heterojunction photodetector decreases by less than 15% even at 10 kHz and remains more than 10% at 1 MHz. In contrast, relative balance of the device without graphene transparent electrode diminishes sharply and approximates to zero at 10 kHz.

**Figure 5:**
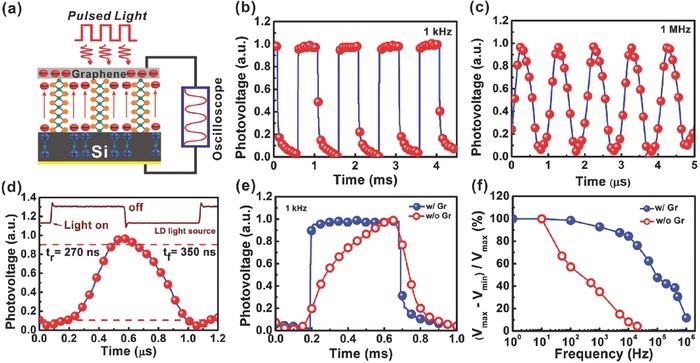
a) Schematic illustration shows the rapid separation and transport of electrons and holes along the in‐plane direction of MoSe_2_ under pulsed light illumination. The electrons were collected by graphene transparent electrode, ensuring the fast transport of electrons to the outside electrical circuit. Variation of photovoltage is monitored by an oscilloscope to determine the response speed. Photoresponse of the self‐powered photodetector to pulsed light illumination with varied frequency of b) 1 kHz, and c) 1 MHz. d) Enlarged photoresponse curve at 1 MHz. Rise time (*t*_r_) and fall time (*t*_f_) are the time intervals between 10% and 90% of peak response. Response curve of the 650 nm LD light source is also shown in the upper part of the figure for comparison. e) Photoresponse of the MoSe_2_/Si heterojunction with and without graphene transparent electrode under pulsed light illumination. f) Relative balance (*V*_max_–*V*_min_)/*V*_max_ versus switching frequency of the devices with and without graphene.

To gain insight into the mechanism of graphene top electrode for carrier collection, device simulation was conducted on the MoSe_2_/Si heterojunction by using a 2D semiconductor simulation package (ISE‐TCAD). **Figure** **6**a shows the simulated total current density distribution of the device with graphene top electrode at a forward bias of +1 V. The current distributes uniformly with high current density in the MoSe_2_ layer with the use of graphene electrode. In contrast, if only side part of the MoSe_2_ layer contacts with Ag electrode, corresponding to the case without graphene top electrode, the current distribution is very inhomogeneous (Figure 6b); only the region closes to the electrode shows higher current density, while the region (I section) away from the electrode has very low current density, indicating the poor carrier collection capability for the device. This result further confirms the important role of graphene transparent electrode in enhancing the device performance.

**Figure 6:**
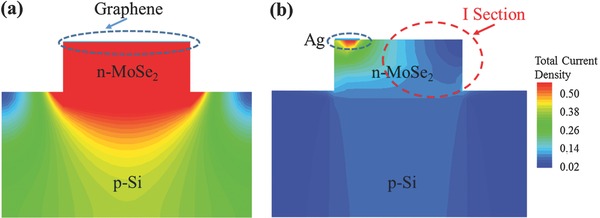
Simulated total current density distributions of the MoSe_2_/Si photodetectors a) with and b) without graphene top electrode at forward bias of +1 V, respectively.

In summary, high‐performance Gr/MoSe_2_/Si heterojunction photodetectors were constructed by depositing MoSe_2_ film with vertically standing layered structure on Si substrate. The heterojunction photodetectors can surpass conventional mono‐/multilayer structured MoSe_2_ photodetectors in terms of high light sensitivity (due to stronger light absorption of the thick film) and ultrafast response speed (due to presence of strong built‐in electric field and short transmit time). The high in‐plane mobility of MoSe_2_ ensures the fast separation and transport of photogenerated carriers from the junction interface to top electrode in the vertically aligned MoSe_2_ film. Moreover, three‐layer graphene was used as transparent electrode to further enhance the carrier collection and thus reduce the recombination at junction interface. As a result, the Gr/MoSe_2_/Si heterojunction photodetectors exhibited outstanding device characteristics with a wide response spectrum range of 365–1310 nm and an ultrafast response speed of ≈270 ns, which represent the best results achieved for MoSe_2_‐based photodetectors thus far. This work unveils the great potential of MoSe_2_/Si heterojunction for high‐performance optoelectronic devices.

## Experimental Section

*Growth of MoSe_2_ Film and Graphene*: MoSe_2_ films were deposited on the Si substrate by RF magnetron sputtering (Kurt J Lesker, PVD 75) using a 2 in. MoSe_2_ target (ZhongNuo Advanced Material (Beijing) Company). The RF power was kept at 50 W with a direct current (DC) bias voltage of 225 V. The pressure in the chamber was 3 mTorr during deposition, and the substrate temperature was controlled to be 400 °C. To increase the quality of MoSe_2_ film, the as‐prepared MoSe_2_ films were annealed in a rapid thermal process system (RTP‐500V) at 800 °C for 30 min. Graphene used here was synthesized by CVD method using a mixed gas of H_2_ (3 sccm) and CH_4_ (50 sccm) as gas source at 1000 °C, and a 25 μm thick copper foil was used as the catalyst substrate.

*Fabrication of Devices*: To fabricated the Gr/MoSe_2_/Si heterojunction photodetectors, a circular photoresist window (*d* = 3 mm) was first defined on the SiO_2_ (300 nm)/p‐type (100) Si (resistivity 1–10 Ω cm^−1^) substrate by photolithography (SUSS, MicroTec‐MJB4), followed by 5% HF etching for 300 s to remove the SiO_2_ in the window. After removing the residual photoresist by acetone, 200 nm MoSe_2_ film was deposited onto the window by sputtering. Afterwards, 50 nm Ag were deposited by e‐beam evaporation (Kurt J Lesker, PVD 75) around the circular window on the MoSe_2_ film using a shadow mask. Three‐layer graphene film was transferred onto the top of the MoSe_2_ film as transparent electrode. 50 nm Au was deposited by e‐beam evaporation onto the rear side of SiO_2_/Si substrate as ohmic contact to Si.

*Material and Device Characterizations*: The thickness and crystal structures of MoSe_2_ film were determined by atomic force microscopy (AFM, Veeco) and X‐ray diffraction (XRD, PANalytical Empyrean), respectively. Raman spectra of the MoSe_2_ film were measured in a LabRAMHR800 Raman microscopy system using 514 nm laser as excitation source. XPS measurements were performed using a monochromatic Al Kα source (1486.6 eV) produced by the XPS system (Kratos AXIS Ultra^DLD^). The XPS data were calibrated by comparing with C 1s peak to assure the correction. The absorption spectra of MoSe_2_ on quartz glass, Si substrate, and MoSe_2_/Si heterojunction were detected by UV–vis spectrometer (Perkin‐Elmer LAMBDA 750) equipped with an integrating sphere. To observe the interfacial structure of heterojunction, the cross‐section structure, element mapping, and line‐scan EDX were characterized by HRTEM (FEI Tecnai G2). The photoresponse of the photodetectors was detected using a semiconductor parameter analyzer (Keithley 4200‐SCS). The light sources were produced by a Xe lamp (CEL‐HXF300) with tunable output power and a monochromator (Zolix Instruments, Omni‐nx I). To measure the NIR photoresponse, 808, 1064, and 1310 nm lasers were also used as light sources (Changchun Laser Optoelectronics Technology, MW‐GX‐808, MW‐GX‐1064, and MW‐GX‐1310). Light intensity is measured by an optical power meter (CEL‐NP2000). The response speed of the photodetector was measured by using a LD (650 nm) driven by a function signal generator (Shengpu, F‐40), and a digital oscilloscope (Tektronix TDS 2012C) was used to monitor the variation of photovoltage.

## Supporting information

As a service to our authors and readers, this journal provides supporting information supplied by the authors. Such materials are peer reviewed and may be re‐organized for online delivery, but are not copy‐edited or typeset. Technical support issues arising from supporting information (other than missing files) should be addressed to the authors.

Supplementary

## References

[1] K. Novoselov , A. K. Geim , S. Morozov , D. Jiang , M. Katsnelson , I. Grigorieva , S. Dubonos , A. Firsov , Nature 2005, 438, 197. 16281030 10.1038/nature04233

[2] A. K. Geim , K. S. Novoselov , Nat. Mater. 2007, 6, 183. 17330084 10.1038/nmat1849

[3] V. W. Brar , M. S. Jang , M. Sherrott , S. Kim , J. J. Lopez , L. B. Kim , M. Choi , H. Atwater , Nano Lett. 2014, 14, 3876. 24874205 10.1021/nl501096s

[4] X. Ling , W. Fang , Y.‐H. Lee , P. T. Araujo , X. Zhang , J. F. Rodriguez‐Nieva , Y. Lin , J. Zhang , J. Kong , M. S. Dresselhaus , Nano Lett. 2014, 14, 3033. 24780008 10.1021/nl404610c

[5] J. Feng , X. Sun , C. Wu , L. Peng , C. Lin , S. Hu , J. Yang , Y. Xie , J. Am. Chem. Soc. 2011, 133, 17832. 21951158 10.1021/ja207176c

[6] Y. Sun , S. Gao , Y. Xie , Chem. Soc. Rev. 2014, 43, 530. 24122032 10.1039/c3cs60231a

[7] Y. Zhou , Y. Nie , Y. Liu , K. Yan , J. Hong , C. Jin , Y. Zhou , J. Yin , Z. Liu , H. Peng , ACS Nano 2014, 8, 1485. 24392815 10.1021/nn405529r

[8] M. Xu , T. Liang , M. Shi , H. Chen , Chem. Rev. 2013, 113, 3766. 23286380 10.1021/cr300263a

[9] E. Yoo , J. Kim , E. Hosono , H.‐S. Zhou , T. Kudo , I. Honma , Nano Lett. 2008, 8, 2277. 18651781 10.1021/nl800957b

[10] G. H. Chen , Y. Q. Yu , K. Zheng , T. Ding , W. L. Wang , Y. Jiang , Q. Yang , Small 2015, 11, 2848. 25703599 10.1002/smll.201403508

[11] Q. H. Wang , K. Kalantar‐Zadeh , A. Kis , J. N. Coleman , M. S. Strano , Nat. Nanotech. 2012, 7, 699. 10.1038/nnano.2012.193 23132225

[12] W. S. Yun , S. Han , S. C. Hong , I. G. Kim , J. Lee , Phys. Rev. B 2012, 85, 033305.

[13] M. Chhowalla , H. S. Shin , G. Eda , L.‐J. Li , K. P. Loh , H. Zhang , Nat. Chem. 2013, 5, 263. 23511414 10.1038/nchem.1589

[14] S.‐L. Li , K. Komatsu , S. Nakaharai , Y.‐F. Lin , M. Yamamoto , X. Duan , K. Tsukagoshi , ACS Nano 2014, 8, 12836. 25470503 10.1021/nn506138y

[15] R. Cheng , D. Li , H. Zhou , C. Wang , A. Yin , S. Jiang , Y. Liu , Y. Chen , Y. Huang , X. Duan , Nano Lett. 2014, 14, 5590. 25157588 10.1021/nl502075n PMC4189621

[16] D. Voiry , H. Yamaguchi , J. Li , R. Silva , D. C. Alves , T. Fujita , M. Chen , T. Asefa , V. B. Shenoy , G. Eda , Nat. Mater. 2013, 12, 850. 23832127 10.1038/nmat3700

[17] H. Li , X. Duan , X. Wu , X. Zhuang , H. Zhou , Q. Zhang , X. Zhu , W. Hu , P. Ren , P. Guo , J. Am. Chem. Soc. 2014, 136, 3756. 24564365 10.1021/ja500069b

[18] H. Fang , S. Chuang , T. C. Chang , K. Takei , T. Takahashi , A. Javey , Nano Lett. 2012, 12, 3788. 22697053 10.1021/nl301702r

[19] A. M. Jones , H. Yu , N. J. Ghimire , S. Wu , G. Aivazian , J. S. Ross , B. Zhao , J. Yan , D. G. Mandrus , D. Xiao , Nat. Nanotech. 2013, 8, 634. 10.1038/nnano.2013.151 23934096

[20] X. Huang , Z. Zeng , H. Zhang , Chem. Soc. Rev. 2013, 42, 1934. 23344899 10.1039/c2cs35387c

[21] C. Ataca , H. Sahin , S. Ciraci , J. Phys. Chem. C 2012, 116, 8983. 10.1103/PhysRevLett.108.126103 22540600

[22] K. F. Mak , C. Lee , J. Hone , J. Shan , T. F. Heinz , Phys. Rev. Lett. 2010, 105, 136805. 21230799 10.1103/PhysRevLett.105.136805

[23] Y. Yoon , K. Ganapathi , S. Salahuddin , Nano Lett. 2011, 11, 3768. 21790188 10.1021/nl2018178

[24] X. Wang , Y. Gong , G. Shi , W. L. Chow , K. Keyshar , G. Ye , R. Vajtai , J. Lou , Z. Liu , E. Ringe , ACS Nano 2014, 8, 5125. 24680389 10.1021/nn501175k

[25] N. Kumar , Q. Cui , F. Ceballos , D. He , Y. Wang , H. Zhao , Nanoscale 2014, 6, 4915. 24671171 10.1039/c3nr06863c

[26] M. Bernardi , M. Palummo , J. C. Grossman , Nano Lett. 2013, 13, 3664. 23750910 10.1021/nl401544y

[27] H. Li , Q. Zhang , X. Duan , X. Wu , X. Fan , X. Zhu , X. Zhuang , W. Hu , H. Zhou , A. Pan , J. Am. Chem. Soc. 2015, 137, 5284. 25871953 10.1021/jacs.5b01594

[28] L. T. L. Lee , J. He , B. Wang , Y. Ma , K. Y. Wong , Q. Li , X. Xiao , T. Chen , Sci. Rep. 2014, 4, 4063. 24525919 10.1038/srep04063 PMC3924216

[29] Y. Shi , C. Hua , B. Li , X. Fang , C. Yao , Y. Zhang , Y. S. Hu , Z. Wang , L. Chen , D. Zhao , Adv. Funct. Mater. 2013, 23, 1832.

[30] S. Larentis , B. Fallahazad , E. Tutuc , Appl. Phys. Lett. 2012, 101, 223104.

[31] V. Pathak , K. Patel , R. Pathak , R. Srivastava , Sol. Energy Mater. Sol. Cells 2002, 73, 117.

[32] B. Shin , Y. Zhu , N. A. Bojarczuk , S. J. Chey , S. Guha , Appl. Phys. Lett. 2012, 101, 053903.

[33] M. Fontana , T. Deppe , A. K. Boyd , M. Rinzan , A. Y. Liu , M. Paranjape , P. Barbara , Sci. Rep. 2013, 3. 10.1038/srep01634 PMC3620663 23567328

[34] O. Lopez‐Sanchez , D. Lembke , M. Kayci , A. Radenovic , A. Kis , Nat. Nanotech. 2013, 8, 497. 10.1038/nnano.2013.100 23748194

[35] C. Zhang , S. Wang , L. Yang , Y. Liu , T. Xu , Z. Ning , A. Zak , Z. Zhang , R. Tenne , Q. Chen , Appl. Phys. Lett. 2012, 100, 243101.

[36] F. Xia , T. Mueller , Y.‐M. Lin , A. Valdes‐Garcia , P. Avouris , Nat. Nanotech. 2009, 4, 839. 10.1038/nnano.2009.292 19893532

[37] X. Wang , Z. Cheng , K. Xu , H. K. Tsang , J.‐B. Xu , Nat. Photon. 2013, 7, 888.

[38] Y. Zhang , T. Liu , B. Meng , X. Li , G. Liang , X. Hu , Q. J. Wang , Nat. Commun. 2013, 4, 1811. 23651999 10.1038/ncomms2830

[39] Z. Sun , H. Chang , ACS Nano 2014, 8, 4133. 24716438 10.1021/nn500508c

[40] Q. Zhang , J. Jie , S. Diao , Z. Shao , Q. Zhang , L. Wang , W. Deng , W. Hu , H. Xia , X. Yuan , ACS Nano 2015, 9, 1561. 25625624 10.1021/acsnano.5b00437

[41] W. Zhang , M.‐H. Chiu , C.‐H. Chen , W. Chen , L.‐J. Li , A. T. S. Wee , ACS Nano 2014, 8, 8653. 25106792 10.1021/nn503521c

[42] B. Liu , L. Chen , G. Liu , A. N. Abbas , M. Fathi , C. Zhou , ACS Nano 2014, 8, 5304. 24749814 10.1021/nn5015215

[43] Y.‐H. Chang , W. Zhang , Y. Zhu , Y. Han , J. Pu , J.‐K. Chang , W.‐T. Hsu , J.‐K. Huang , C.‐L. Hsu , M.‐H. Chiu , ACS Nano 2014, 8, 8582. 25094022 10.1021/nn503287m

[44] C. Fan , Q. Yue , J. Yang , Z. Wei , S. Yang , J. Li , Appl. Phys. Lett. 2014, 104, 202105.

[45] J. Xia , X. Huang , L.‐Z. Liu , M. Wang , L. Wang , B. Huang , D.‐D. Zhu , J.‐J. Li , C.‐Z. Gu , X.‐M. Meng , Nanoscale 2014, 6, 8949. 24965908 10.1039/c4nr02311k

[46] H. Xu , X. Xiao , X. Li , Y. Hu , Z. Li , T. Chu , Y. Yu , J. Yu , Opt. Express 2012, 20, 15093. 22772206 10.1364/OE.20.015093

[47] J. Xing , K. Jin , M. He , H. Lu , G. Liu , G. Yang , J. Phys. D: Appl. Phys. 2008, 41, 195103.

[48] Y. Q. Bie , Z. M. Liao , H. Z. Zhang , G. R. Li , Y. Ye , Y. B. Zhou , J. Xu , Z. X. Qin , L. Dai , D. P. Yu , Adv. Mater. 2011, 23, 649. 21274914 10.1002/adma.201003156

[49] X. Duan , C. Wang , J. C. Shaw , R. Cheng , Y. Chen , H. Li , X. Wu , Y. Tang , Q. Zhang , A. Pan , Nat. Nanotech. 2014, 9, 1024. 10.1038/nnano.2014.222 PMC12049235 25262331

[50] G. W. Shim , K. Yoo , S.‐B. Seo , J. Shin , D. Y. Jung , I.‐S. Kang , C. W. Ahn , B. J. Cho , S.‐Y. Choi , ACS Nano 2014, 8, 6655. 24987802 10.1021/nn405685j

[51] C. Fan , Z. Wei , S. Yang , J. Li , RSC Adv. 2014, 4, 775.

[52] A. Abderrahmane , P. J. Ko , T. V. Thu , S. Ishizawa , T. Takamura , A. Sandhu , Nanotechnology 2014, 25, 365202. 25140619 10.1088/0957-4484/25/36/365202

[53] D.‐S. Tsai , K.‐K. Liu , D.‐H. Lien , M.‐L. Tsai , C.‐F. Kang , C.‐A. Lin , L.‐J. Li , J.‐H. He , Acs Nano 2013, 7, 3905. 23590667 10.1021/nn305301b

[54] X. An , F. Liu , Y. J. Jung , S. Kar , Nano Lett. 2013, 13, 909. 23350824 10.1021/nl303682j

[55] H. Li , G. Lu , Y. Wang , Z. Yin , C. Cong , Q. He , L. Wang , F. Ding , T. Yu , H. Zhang , Small 2013, 9, 1974. 23281258 10.1002/smll.201202919

[56] Q. Yu , L. A. Jauregui , W. Wu , R. Colby , J. Tian , Z. Su , H. Cao , Z. Liu , D. Pandey , D. Wei , Nat. Mater. 2011, 10, 443. 21552269 10.1038/nmat3010

[57] X. Lu , M. I. B. Utama , J. Lin , X. Gong , J. Zhang , Y. Zhao , S. T. Pantelides , J. Wang , Z. Dong , Z. Liu , Nano Lett. 2014, 14, 2419. 24678857 10.1021/nl5000906

[58] Z. Mutlu , D. Wickramaratne , H. H. Bay , Z. J. Favors , M. Ozkan , R. Lake , C. S. Ozkan , Phys. Status Solidi A 2014, 211, 2671.

[59] J. Xia , X. Huang , L. Liu , M. Wang , L. Wang , B. Huang , D. Zhu , J. J. Li , C.‐Z. Gu , X. Meng , Nanoscale 2014, 6, 8949. 24965908 10.1039/c4nr02311k

[60] C. Lee , H. Yan , L. E. Brus , T. F. Heinz , J. Hone , S. Ryu , ACS Nano 2010, 4, 2695. 20392077 10.1021/nn1003937

[61] D. Kong , H. Wang , J. J. Cha , M. Pasta , K. J. Koski , J. Yao , Y. Cui , Nano Lett. 2013, 13, 1341. 23387444 10.1021/nl400258t

[62] H. Tang , K. Dou , C.‐C. Kaun , Q. Kuang , S. Yang , J. Mater. Chem. A 2014, 2, 360.

[63] W. e. A. Abdallah , A. Nelson , J. Mater. Sci. 2005, 40, 2679.

[64] J. C. Shaw , H. Zhou , Y. Chen , N. O. Weiss , Y. Liu , Y. Huang , X. Duan , Nano Res. 2014, 7, 511.

[65] R.‐S. Chen , C.‐C. Tang , W.‐C. Shen , Y.‐S. Huang , Nanotechnology 2014, 25, 415706. 25249412 10.1088/0957-4484/25/41/415706

[66] M. Buscema , J. O. Island , D. J. Groenendijk , S. I. Blanter , G. A. Steele , H. S. van der Zant , A. Castellanos‐Gomez , Chem. Soc. Rev. 2015, 44, 3691. 25909688 10.1039/c5cs00106d

[67] X. Zhang , X. Zhang , X. Zhang , Y. Zhang , L. Bian , Y. Wu , C. Xie , Y. Han , Y. Wang , P. Gao , J. Mater. Chem. 2012, 22, 22873.

[68] C. Xie , B. Nie , L. Zeng , F.‐X. Liang , M.‐Z. Wang , L. Luo , M. Feng , Y. Yu , C.‐Y. Wu , Y. Wu , ACS Nano 2014, 8, 4015. 24665986 10.1021/nn501001j

[69] Ş. Karataş , Ş. Altındal , A. Türüt , A. Özmen , Appl. Surf. Sci. 2003, 217, 250.

[70] Y.‐Q. Yu , L.‐B. Luo , Z.‐F. Zhu , B. Nie , Y.‐G. Zhang , L.‐H. Zeng , Y. Zhang , C.‐Y. Wu , L. Wang , Y. Jiang , CrystEngComm 2013, 15, 1635.

[71] M. S. Choi , D. Qu , D. Lee , X. Liu , K. Watanabe , T. Taniguchi , W. J. Yoo , ACS Nano 2014, 8, 9332. 25131298 10.1021/nn503284n

