# Supplementary material for: Ultrafast, Broadband Photodetector Based on MoSe2/Silicon Heterojunction with Vertically Standing Layered Structure Using Graphene as Transparent Electrode
Source: Adv Sci (Weinh). 2016 Jul 5;3(11):1600018. doi: 10.1002/advs.201600018 (PMC5102659; doi:10.1002/advs.201600018)
Supplement: Supplementary file 1 — Supplementary [file ADVS-3-0p-s001.pdf]

Copyright WILEY-VCH Verlag GmbH & Co. KGaA, 69469 Weinheim, Germany, 2016.

Supporting Information

for *Adv. Sci.,* DOI: 10.1002/advs.201600018

Ultrafast, Broadband Photodetector Based on $MoSe_2$/Silicon Heterojunction with Vertically Standing Layered Structure Using Graphene as Transparent Electrode

Jie Mao, Yongqiang Yu, Liu Wang, Xiujuan Zhang, Yuming Wang, Zhibin Shao, and Jiansheng Jie**

Copyright WILEY-VCH Verlag GmbH & Co. KGaA, 69469 Weinheim, Germany, 2013.

Supporting Information

Ultrafast, Broadband Photodetector Based on $MoSe_2$/Silicon Heterojunction with Vertically Standing Layered Structure Using Graphene as Transparent Electrode

Jie Mao,[#] Yongqiang Yu,[#] Liu Wang, Xiujuan Zhang, Yuming Wang, Zhibing Shao, Jiansheng Jie**

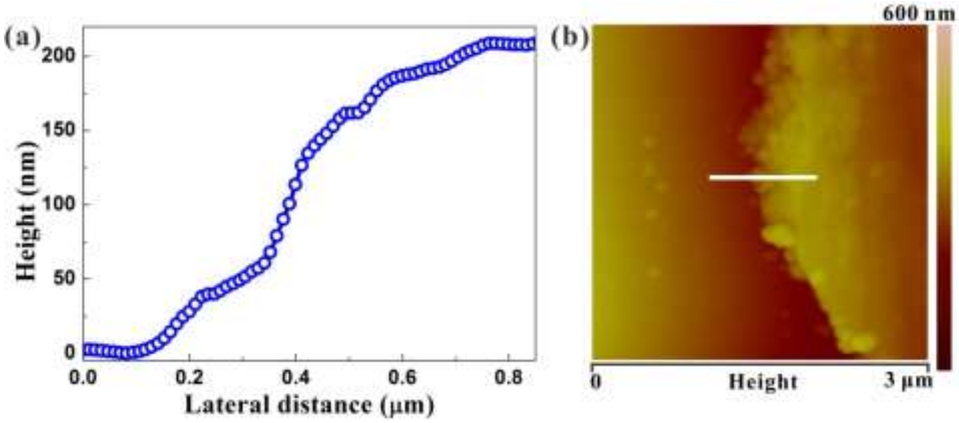

Figure S1. AFM characterization of the n-type $MoSe_2$ film grown on p-type Si substrate. (a) Thickness scan along the white line across the $MoSe_2$/Si interface in (b).

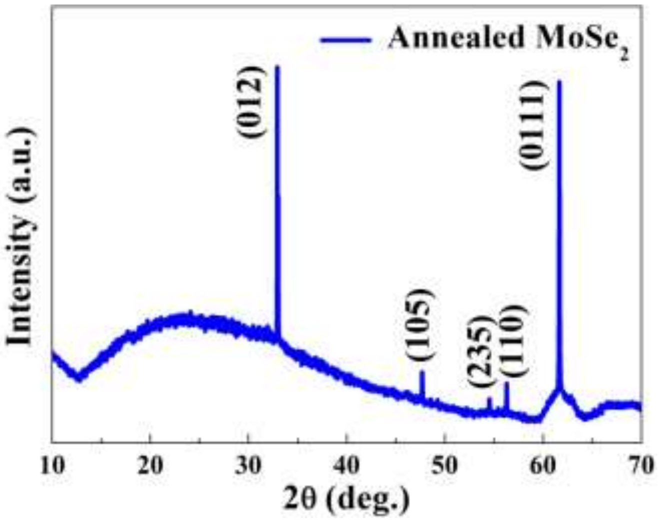

Figure S2. XRD pattern of annealed $MoSe_2$ film, indicating trigonal phase of the $MoSe_2$ film (JCPDS Card No. 20-0757).

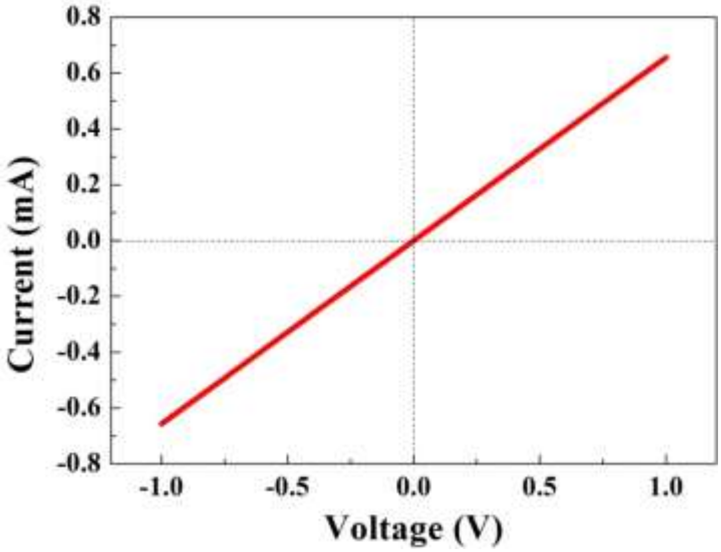

Figure S3. *I-V* characteristics of the Ag/$MoSe_2$/Ag stacking structure. The linear shape of the curve indicats the ohmic contact of Ag electrode with $MoSe_2$ film.

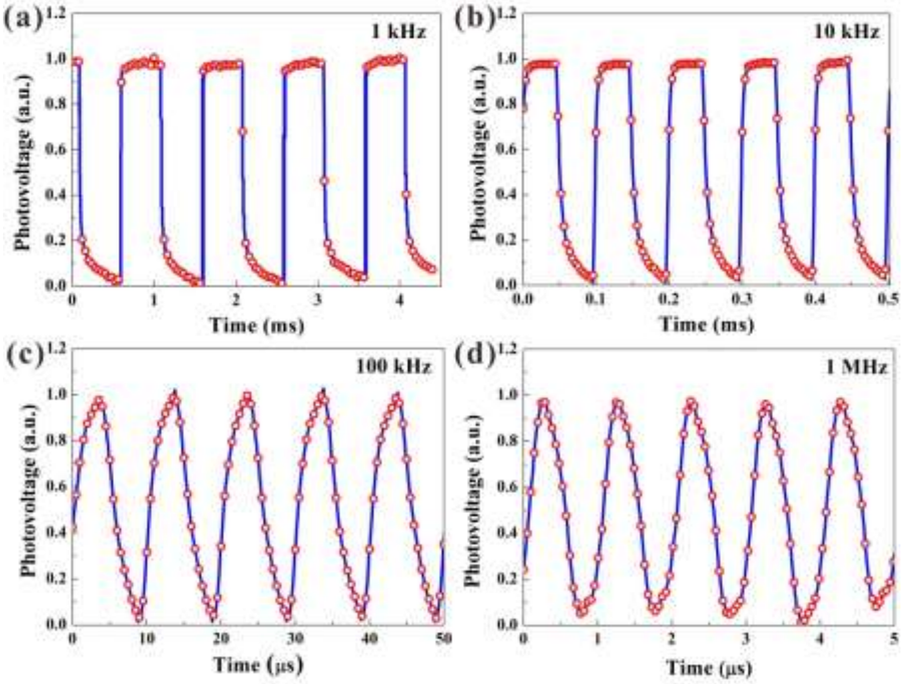

Figure S4. Photoresponse of the Gr/$MoSe_2$/Si photodetector to pulsed light illumination with varied frequency from 1 kHz to1 MHz.
